# Using the Cochrane Central Register of Controlled Trials to identify clinical trial registration is insufficient: a cross-sectional study

**DOI:** 10.1186/s12874-020-01083-y

**Published:** 2020-07-25

**Authors:** Masahiro Banno, Yasushi Tsujimoto, Yuki Kataoka

**Affiliations:** 1 Department of Psychiatry, Seichiryo Hospital, Tsurumai 4-16-27, Showa-ku, Nagoya, 466-0064 Japan; 2 Department of Psychiatry, Nagoya University Graduate School of Medicine, Tsurumai-cho 65, Showa-ku, Nagoya, 466-8560 Japan; 3 Systematic Review Workshop Peer Support Group (SRWS-PSG), Osaka, Japan; 4 Department of Healthcare Epidemiology, Graduate School of Medicine and Public Health, Kyoto University, Yoshida Konoe-cho, Sakyo-ku, Kyoto, 606-8501 Japan; 5 Department of Nephrology and Dialysis, Kyoritsu Hospital, Chuo-cho 16-5, Kawanishi, 666-0016 Japan; 6 Hospital Care Research Unit, Hyogo Prefectural Amagasaki General Medical Center, Higashinaniwa-cho 2-17-77, Amagasaki, 660-8550 Japan; 7 Department of Respiratory Medicine, Hyogo Prefectural Amagasaki General Medical Center, Higashinaniwa-cho 2-17-77, Amagasaki, 660-8550 Japan

**Keywords:** Cochrane central register of controlled trials, Clinical trial registration, Sensitivity, Research on research, Meta-research, Meta-epidemiological study

## Abstract

**Background:**

While conducting systemic reviews, searching for ongoing or unpublished trials is critical to address publication bias. As of April 2019, records of ongoing or unpublished randomized and/or quasi-randomized controlled trials registered in the International Clinical Trials Registry Platform (ICTRP) and ClinicalTrials.gov are available in the Cochrane Central Register of Controlled Trials (CENTRAL). These records registered in CENTRAL include studies published since the inception of ICTRP and ClinicalTrials.gov. Whether systematic reviewers can search CENTRAL to identify ongoing or unpublished trials instead of ICTRP and ClinicalTrials.gov is unknown.

**Methods:**

This was a cross-sectional study. A consecutive sample of ongoing or unpublished studies published from June 1, 2019 to December 27, 2019 was selected from the Cochrane Reviews.

The sensitivity and the number needed to read (NNR) were assessed from among the studies selected from CENTRAL instead of ICTRP and ClinicalTrials.gov and also assessed the characteristics of studies not identified by searching CENTRAL.

**Results:**

In total, 247 records from 50 Cochrane reviews were included; of these, 200 were identified by searching CENTRAL, whereas the remaining 47 records were not. The sensitivity of searching CENTRAL was 0.81 (95% confidence interval [CI]: 0.76, 0.85). The NNR was 115 (95% CI: 101, 133). The 47 unidentified studies were registered through ClinicalTrials.gov or ICTRP. Sixteen unidentified studies were not indexed in CENTRAL.

**Conclusions:**

For systematic reviewers, searching CENTRAL could not substitute for searching ClinicalTrials.gov and/or ICTRP. Systematic reviewers should not only search CENTRAL but also ICTRP and ClinicalTrials.gov to identify unpublished trials.

**Trial registration:**

A pre-specified protocol was applied to conduct this study. The study was registered in the University Hospital Medical Information Network Clinical Trials Registry (UMIN-CTR). Trial registration number: UMIN000038981.

## Background

The identification of ongoing or unpublished studies when performing systematic reviews is essential to avoid publication bias [1]. The Cochrane Handbook and previous studies suggest that systematic reviewers search both the International Clinical Trials Registry Platform (ICTRP) and ClinicalTrials.gov for ongoing or unpublished studies [1–4]. As of April 2019, records of ongoing or unpublished randomized controlled trials (RCTs) and quasi-randomized controlled trials (quasi-RCTs) since the inception of ICTRP and ClinicalTrials.gov have been included in the Cochrane Central Register of Controlled Trials (CENTRAL) [5], and newly submitted records are indexed in CENTRAL on a monthly basis [6].

Systematic reviews are time and resource intensive research [7]. Cochrane authors are often able to use the specialized registers to identify relevant studies, including trial registration, whereas non-Cochrane reviewers cannot access the same. Reducing the effort to search ICTRP and ClinicalTrials.gov will save time in the completion of non-Cochrane systematic reviews. We aimed to investigate whether systematic reviewers can use CENTRAL to identify ongoing or unpublished trials, although it had been not intended to be used according to standard practice, because reducing the time required to conduct a systematic review by as much as possible is important [7]. The aim of this study was to answer the question 1.“Can systematic reviewers rely on searching CENTRAL alone to identify ongoing and unpublished studies rather than conducting parallel searches of trial registries?” and 2.Examining the sensitivity and the number needed read (NNR) using CENTRAL to identify ongoing or unpublished studies.

## Methods

This study was registered in the University Hospital Medical Information Network Clinical Trials Registry (UMIN-CTR) (registration number UMIN000038981). Moreover, before study onset, the protocol was uploaded to medRxiv (registration number: medRxiv 2019.12.26.19014274) [8].

### Study design

**Fig. 1:**
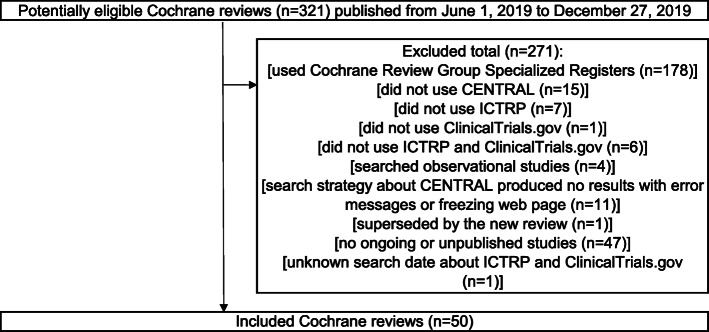
Inclusion criteria for screening the Cochrane Reviews. Abbreviations: CENTRAL, Cochrane Central Register of Controlled Trials; ICTRP, International Clinical Trials Registry Platform

**Fig. 2:**
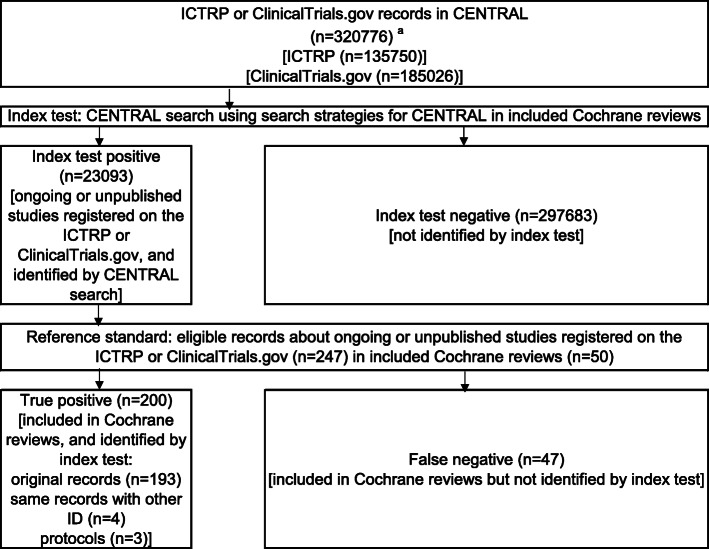
Search strategy for identification of registration records. Abbreviations: CENTRAL, Cochrane Central Register of Controlled Trials; ICTRP, International Clinical Trials Registry Platform; ID, Identifier. ^a^ The number of ICTRP or ClinicalTrials.gov records in CENTRAL was estimated by limiting the publication year up to and including 2019

This was a cross-sectional study. The STROBE (STrengthening the Reporting of OBservational studies in Epidemiology) Statement (2007) were adhered for reporting cross-sectional studies based on the STROBE checklist for cross-sectional studies (Appendix S1) [9]. The guideline was only partially adhered to as this study was ‘research on research’ investigating in cross-sectional design, rather than a clinical cross-sectional study, which is the main target of the guideline. A consecutive sample of ongoing or unpublished records was selected from the Cochrane Reviews. These records were considered the reference standard before determining whether they were also identified by the CENTRAL search. The concept and design of this study are presented in Figs. 1 and 2.

### Eligibility criteria

All records on ongoing or unpublished studies registered in the ICTRP or ClinicalTrials.gov in the Cochrane reviews published from June 1, 2019, to December 27, 2019 were included. Cochrane reviews that did not use CENTRAL, ICTRP, or ClinicalTrials.gov were omitted from the study. We also excluded Cochrane reviews, which searched Cochrane Review Group Specialized Registers, because they were not publicly available. Cochrane reviews that included studies other than controlled trials were also omitted as CENTRAL only indexes controlled studies [5]. In this study, “Cochrane reviews records” were defined as ongoing or unpublished studies included in each eligible Cochrane reviews. “CENTRAL records” were defined as ClinicalTrials.gov or ICTRP records obtained from the CENTRAL search presented in each eligible Cochrane reviews. “Cochrane reviews/CENTRAL records” were defined as Cochrane reviews records that were identified by the CENTRAL search. To calculate the sensitivity of searching CENTRAL to identify Cochrane reviews records, all CENTRAL records were included in the present study. For eligible records, the following characteristics were extracted: trial identifying number, and year of registration.

### Index test and reference standard

The index test was the search of CENTRAL. CENTRAL was manually searched with the search strategy presented in each Cochrane reviews in the limited publication year that corresponded to its search year. The CENTRAL searches were performed from January 2020 to March 2020.

The reference standard was the Cochrane reviews records. One review author (MB) retrieved the citations, which were confirmed by one of two authors (YT and YK). Disagreements between authors were resolved through discussion. Cochrane reviews were selected as the primary data source as these are performed following rigorous methods detailed in the Cochrane Handbook and are expected to be available with sufficient search strategy to perform a comprehensive CENTRAL search [1].

### How was the sample of reviews obtained?

We obtained eligible Cochrane reviews through CENTRAL.

### Sample size

A suitable sample size was not calculated as this was an exploratory study. Cochrane reviews published from June 1, 2019, to December 27, 2019 were selected.

### Data analysis

The primary outcome was sensitivity, with a 95% confidence interval (CI), that searching CENTRAL would discover all Cochrane reviews records. Sensitivity was estimated by dividing the number of Cochrane reviews/CENTRAL records by the number of Cochrane reviews records [1]. The 95% CI in Wilson score interval was estimated without continuity correction because this method is considered to be more accurate than the conventional method [10].

A pre-specified subgroup analysis regarding the primary outcome was performed as follows: 1) the type of intervention in the Cochrane reviews (pharmacological, non-pharmacological, or both) and 2) the Cochrane reviews version (first or updated). Ad-hoc sensitivity analysis was performed to exclude Cochrane reviews that included observational studies, although they were excluded in the study methods. Ad-hoc sensitivity analysis was also performed to exclude Cochrane reviews that searched trial registries other than ClinicalTrials.gov or ICTRP.

The secondary outcome was the NNR with 95% CI. The NNR is a metric of how many records in a database need to be read to discover one of adequate clinical quality and relevance [11]. We estimated the NNR and 95% CI as the following proportion and 95% CI: In the numerator was the CENTRAL records, while the denominator had Cochrane reviews/CENTRAL records [1]. The number of CENTRAL records (ICTRP or ClinicalTrials.gov records in CENTRAL) was estimated by limiting the publication year up to and including 2019 as shown in Fig. 2. The numbers of records that were included Cochrane reviews but were not discovered by searching CENTRAL were also determined.

The following characteristics of studies not identified by searching CENTRAL were reported: year of registration; registries; whether studies included observational studies; whether studies were indexed in CENTRAL; whether studies were included in Cochrane reviews that searched other trial registers excluding ClinicalTrials.gov or ICTRP; whether studies were included in Cochrane reviews; and which types of intervention were used, including pharmacological, non-pharmacological, and both pharmacological and non-pharmacological. We also investigated who created the search strategies in the Cochrane reviews if we could not identify all ongoing or unpublished studies included in the reviews by searching CENTRAL. All statistical analyses were performed in Stata V.15.1 (StataCorp LLC, College Station, Texas, United States of America) [12].

### Ethics

Ethical approval was not required as this study performed ‘research on research’.

## Results

### Selection process

Figure 1 details a flow diagram of the Cochrane reviews selection. Of the 321 Cochrane reviews identified, 271 were excluded. In total, 50 Cochrane reviews were included. The study details of these 50 Cochrane reviews are detailed in Appendix S2.

Figure 2 details a flow diagram for identifying registration records from CENTRAL. The CENTRAL searches were performed using the search strategies detailed in the individual included Cochrane reviews (index test), with a total of 23,093 CENTRAL records obtained (index test positive). The index test positive list was screened using the 247 Cochrane reviews records (reference standard). Finally, 200 Cochrane reviews/CENTRAL records were obtained (true positive). In total, 47 records were included in the Cochrane reviews but not identified by the index test (false negative). The details of the 47 unidentified studies are shown in Table S1.

### The sensitivity of using CENTRAL to discover Cochrane reviews records

The sensitivity, with 95% CI, of searching CENTRAL to discover Cochrane reviews records was 0.81 [95% CI: 0.76, 0.85]. The results of the subgroup analyses were as follows: 1) the type of intervention on Cochrane reviews (the sensitivity of pharmacological intervention in 21 Cochrane reviews was 0.89 [95% CI: 0.82, 0.94] and the sensitivity of non-pharmacological intervention in 27 Cochrane reviews was 0.75 [95% CI: 0.67, 0.81]) and the sensitivity of pharmacological and non-pharmacological intervention in two Cochrane reviews was 0.67 [95% CI: 0.30, 0.90]; 2) the version of Cochrane reviews (the sensitivity of the first version in 35 Cochrane reviews was 0.79 [95% CI: 0.72, 0.85] and sensitivity of the updated version in 15 Cochrane reviews was 0.84 [95% CI: 0.75, 0.90]). The sensitivity analysis excluding four Cochrane reviews that included observational studies, despite stating their exclusion in the study methods, demonstrated a sensitivity of 0.88 [95% CI: 0.82, 0.91]. The final sensitivity analysis excluding seven Cochrane reviews that had searched trial registries other than ClinicalTrials.gov or ICTRP, demonstrated a sensitivity of 0.88 [95% CI: 0.82, 0.92].

### The NNR of searching CENTRAL to discover Cochrane reviews records

The NNR, with 95% CI, of searching CENTRAL to discover Cochrane reviews records was 115 (95% CI: 101, 133).

### Characteristics of the studies not identified by searching CENTRAL

In total, 19 (39.5%) of 50 Cochrane reviews included at least one study that was not identified by searching CENTRAL. The characteristics of these 47 unidentified studies are detailed in Table 1. However, all of these unidentified studies were registered in ClinicalTrials.gov or ICTRP; most (33 of 47) of the unidentified studies were registered in ClinicalTrials.gov. We found no records from registers other than ClinicalTrials.gov or ICTRP in Cochrane reviews that searched trial registries other than ClinicalTrials.gov or ICTRP. Sixteen (34%) were not indexed in CENTRAL. Of the 16 not indexed in CENTRAL, six were RCTs. Information specialists were involved in creation of the search strategies for 17 (89%) Cochrane reviews, and systematic review authors did this for 2 (11%) Cochrane reviews.

**Table 1:** Characteristics of studies not identified by searching CENTRAL (*N* = 47)

Category	Subcategory	Number (percentage)
Year of registration	2010	2 (4)
	2011	2 (4)
	2012	4 (9)
	2013	3 (6)
	2014	6 (13)
	2015	8 (17)
	2016	7 (15)
	2017	9 (19)
	2018	3 (6)
	2019	3 (6)
Registries	ANZCTR	4 (9)
	ClinicalTrials.gov	33 (70)
	ChiCTR	1 (2)
	IRCT	4 (9)
	ISRCTN	2 (4)
	JPRN	1 (2)
	NTR	1 (2)
	ReBEC	1 (2)
Observational studies ^a^	Yes	10 (21)
	No	37 (79)
Indexed in CENTRAL	Yes	31 (66)
	No	16 (34)
Included in Cochrane reviews that searched trial registries other than ClinicalTrials.gov or ICTRP	Yes	6 (13)
	No	41 (87)
Included in Cochrane reviews which types of intervention were	Pharmacological	12 (26)
	Non-pharmacological	33 (70)
	Both	2 (4)

*Abbreviations*: *CENTRAL* Cochrane Central Register of Controlled Trials, *ANZCTR* Australian New Zealand Clinical Trials Registry, *ChiCTR* Chinese Clinical Trial Registry, *ICTRP* International Clinical Trials Registry Platform, *IRCT* Iranian Registry of Clinical Trials, *ISRCTN* International Standard Randomized Controlled Trial Number Register, *JPRN* Japan Primary Registries Network, *NTR* The Netherlands National Trial Register, *ReBEC* Brazilian Clinical Trials Registry

^a^ Cochrane reviews whose inclusion criteria were limited to controlled trials were included. However, some of these Cochrane reviews also included observational studies as ongoing or unpublished studies

## Discussion

### Brief summary of the main findings

First, this study investigated whether searching CENTRAL alone instead of searching ICTRP and ClinicalTrials.gov was sufficient to identify ongoing or unpublished clinical trial registrations. The sensitivity of searching CENTRAL to discover Cochrane reviews records was 0.81, suggesting that it could not be substituted for searching ClinicalTrials.gov and ICTRP.

### Results in relation to prior studies

These results demonstrating that searching CENTRAL alone is insufficient for identifying ongoing or unpublished clinical trials are similar to those of previous studies that have shown that searching the ICTRP, which included ClinicalTrials.gov, does not always retrieve all ClinicalTrials.gov records [1, 3, 4]. A previous study on the comprehensiveness of 16 database searches to identify all studies included in a systematic review of the effectiveness of an extensive range of interventions for managing frozen shoulder, or painful, persistent stiffness of the shoulder joint reported that a sensitivity and NNR of searching CENTRAL of 76% and 25, respectively [13]. Although the previous study did not focus on ongoing or unpublished studies, the sensitivity of searching CENTRAL was similar to the results of the present study, indicating that searching a single database may not be comprehensive [13]. However, the NNR in the previous study was smaller than that in the present study [13], which could be because it included both published and unpublished studies on shoulder joint interventions [13].

### Why searching CENTRAL alone might be insufficient for identifying ongoing or unpublished studies

The data presented here suggest that trial registries that included missing records in CENTRAL varied.

To some extent, the inclusion or exclusion of observational studies can explain why some ongoing or unpublished studies were not identified by searching CENTRAL, as it does not include observational studies [5]. The inclusion criteria for Cochrane reviews was limited to controlled trials, however, several of these also included observational studies [14–17]. Although excluding such Cochrane reviews improved the overall sensitivity, it still remained unsatisfactory. Such records that are included in ClinicalTrials.gov or ICTRP may not be indexed in CENTRAL due to errors arising during the screening process, either by the Cochrane RCT machine classifier or Cochrane Crowd (manual screening) [5], or because search strategies about CENTRAL might be suitable for searching for MEDLINE and EMBASE records, but not for searching for ICTRP and ClinicalTrials.gov records. Searching alternative trial registries may also explain ongoing or unpublished studies that were not identified; a direct search of the primary registries of the ICTRP may identify larger ongoing or unpublished studies that are not indexed in the ICTRP itself. For example, it has been suggested that certain search terms identified more studies in ClinicalTrials.gov, one of the primary registries in the ICTRP, than in the ICTRP itself [18].

The subgroup analyses presented here consistently showed that searching CENTRAL alone was not sufficient to identify unpublished or ongoing studies. The sensitivity of searching CENTRAL was higher in Cochrane reviews on pharmacological interventions compared to those on nonpharmacological interventions; however, the search still could not be considered to be comprehensive.

It is unlikely that the version of Cochrane reviews underlies why ongoing or unpublished studies were not identified by CENTRAL.

Furthermore, the use of additional sources by some authors, such as searching reference lists of eligible studies in Cochrane reviews, citation search for eligible studies in Cochrane reviews, contacting authors to identify unpublished studies, etc., may also explain why ongoing or unpublished studies were not identified.

Information specialists were involved in creation of the search strategies in most of Cochrane reviews. Therefore, the quality of search strategies for CENTRAL would be high.

### Implications for researchers

The data showing that searching CENTRAL alone was not comprehensive, indicated that researchers should search both ClinicalTrials.gov and ICTRP to identify ongoing or unpublished studies.

### Limitations

The present study has several limitations. Firstly, the focus was Cochrane reviews; thus, the results may not be applicable to non-Cochrane reviews. Non-Cochrane reviews have been shown to search trial registries less often than Cochrane reviews [19].

Secondly, these results cannot be applied to systematic reviews including observational studies such as prognostic reviews or diagnostic test accuracy reviews as CENTRAL includes only controlled trials.

Furthermore, based on the perspective of an appropriate search, the setting of Cochrane reviews as a reference standard may not be appropriate. For example, search strategies about CENTRAL might be incomprehensive, even if the Cochrane information specialists searched, and there might be eligible ongoing studies that were not included in the Cochrane reviews.

Finally, the search strategies used in the included Cochrane reviews were different for CENTRAL, ICTRP, or ClinicalTrials.gov, respectively. However, it was unlikely that the search strategies for CENTRAL had lower comprehensiveness as compared to those of ICTRP or ClinicalTrials.gov and that CENTRAL search quality was low, as Cochrane information specialists made search strategies in terms of their search expertise [1].

## Conclusions

Searching CENTRAL alone instead of searching ICTRP and ClinicalTrials.gov was insufficient to identify ongoing or unpublished clinical trials. The findings of this study suggest that systematic reviewers should not search CENTRAL alone but also ClinicalTrials.gov and ICTRP to identify ongoing or unpublished studies.

## Supplementary information

**Additional file 1: Table S1.** The details of the unidentified studies through searching CENTRAL (*N* = 47). Abbreviations: CENTRAL, Cochrane Central Register of Controlled Trials; ID, Identifier; ANZCTR, Australian New Zealand Clinical Trials Registry; ChiCTR, Chinese Clinical Trial Registry; ICTRP, International Clinical Trials Registry Platform; IRCT, Iranian Registry of Clinical Trials; ISRCTN, International Standard Randomized Controlled Trial Number Register; JPRN, Japan Primary Registries Network; NTR, The Netherlands National Trial Register; ReBEC, Brazilian Clinical Trials Registry.

**Additional file 2: Appendix S1.** STROBE checklist for cross-sectional studies. Abbreviations: STROBE, STrengthening the Reporting of OBservational studies in Epidemiology.

**Additional file 3: Appendix S2**. Reference list of included Cochrane Reviews.

## Data Availability

The datasets generated during and/or analyzed during the current study are available from the corresponding author on reasonable request.

## Abbreviations

MB: Masahiro Banno
YT: Yasushi Tsujimoto
YK: Yuki Kataoka
RCTs: Randomized controlled trials
quasi-RCTs: Quasi-randomized controlled trials
ICTRP: International Clinical Trials Registry Platform
CENTRAL: Cochrane Central Register of Controlled Trials
NNR: Number needed to read
CI: Confidence interval
UMIN-CTR: University Hospital Medical Information Network Clinical Trials Registry
STROBE: Strengthening the Reporting of Observational Studies in Epidemiology
SRWS-PSG: Systematic Review Workshop Peer Support Group
