# Supplementary material for: Using the Cochrane Central Register of Controlled Trials to identify clinical trial registration is insufficient: a cross-sectional study
Source: BMC Med Res Methodol. 2020 Jul 25;20:200. doi: 10.1186/s12874-020-01083-y (PMC7382846; doi:10.1186/s12874-020-01083-y)
Supplement: Supplementary file 2 — Additional file 2: Appendix S1. STROBE checklist for cross-sectional studies. Abbreviations: STROBE, STrengthening the Reporting of OBservational studies in Epidemiology. [file 12874_2020_1083_MOESM2_ESM.docx]

STROBE Statement—Checklist of items that should be included in reports of ***cross-sectional studies***

|  | Item No | Recommendation |
| --- | --- | --- |
| **Title and abstract** | 1 | (*a*) Indicate the study’s design with a commonly used term in the title or the abstract  Page 1, page 3 |
|  |  | (*b*) Provide in the abstract an informative and balanced summary of what was done and what was found  Page 3, page 4 |
| Introduction | | |
| Background/rationale | 2 | Explain the scientific background and rationale for the investigation being reported  Page 5, page 6 |
| Objectives | 3 | State specific objectives, including any prespecified hypotheses  Page 5, page 6 |
| Methods | | |
| Study design | 4 | Present key elements of study design early in the paper  Page 6 |
| Setting | 5 | Describe the setting, locations, and relevant dates, including periods of recruitment, exposure, follow-up, and data collection  Page 6, page 7, page 8 |
| Participants | 6 | (*a*) Give the eligibility criteria, and the sources and methods of selection of participants  Page 6, page 7 |
| Variables | 7 | Clearly define all outcomes, exposures, predictors, potential confounders, and effect modifiers. Give diagnostic criteria, if applicable  Page 8, page 9 |
| Data sources/ measurement | 8* | For each variable of interest, give sources of data and details of methods of assessment (measurement). Describe comparability of assessment methods if there is more than one group  Page 8, page 9 |
| Bias | 9 | Describe any efforts to address potential sources of bias  Not applicable |
| Study size | 10 | Explain how the study size was arrived at  Page 8 |
| Quantitative variables | 11 | Explain how quantitative variables were handled in the analyses. If applicable, describe which groupings were chosen and why  Not applicable |
| Statistical methods | 12 | (*a*) Describe all statistical methods, including those used to control for confounding  Page 8, page 9, page 10 |
|  |  | (*b*) Describe any methods used to examine subgroups and interactions  Page 8, page 9 |
|  |  | (*c*) Explain how missing data were addressed  Not applicable |
|  |  | (*d*) If applicable, describe analytical methods taking account of sampling strategy  Not applicable |
|  |  | (*e*) Describe any sensitivity analyses  Page 8, page 9 |
| Results | | |
| Participants | 13* | (a) Report numbers of individuals at each stage of study—eg numbers potentially eligible, examined for eligibility, confirmed eligible, included in the study, completing follow-up, and analysed  Page 10 |
|  |  | (b) Give reasons for non-participation at each stage  Page 10 |
|  |  | (c) Consider use of a flow diagram  Figure 1, Figure 2 |
| Descriptive data | 14* | (a) Give characteristics of study participants (eg demographic, clinical, social) and information on exposures and potential confounders  Not applicable |
|  |  | (b) Indicate number of participants with missing data for each variable of interest  Not applicable |
| Outcome data | 15* | Report numbers of outcome events or summary measures  Page 10, page 11 |
| Main results | 16 | (*a*) Give unadjusted estimates and, if applicable, confounder-adjusted estimates and their precision (eg, 95% confidence interval). Make clear which confounders were adjusted for and why they were included  Page 10, page 11 |
|  |  | (*b*) Report category boundaries when continuous variables were categorized  Not applicable |
|  |  | (*c*) If relevant, consider translating estimates of relative risk into absolute risk for a meaningful time period  Not applicable |
| Other analyses | 17 | Report other analyses done—eg analyses of subgroups and interactions, and sensitivity analyses  Page 11 |
| Discussion | | |
| Key results | 18 | Summarise key results with reference to study objectives  Page 14 |
| Limitations | 19 | Discuss limitations of the study, taking into account sources of potential bias or imprecision. Discuss both direction and magnitude of any potential bias  Page 17 |
| Interpretation | 20 | Give a cautious overall interpretation of results considering objectives, limitations, multiplicity of analyses, results from similar studies, and other relevant evidence  Page 14, page 15, page 16, page 17 |
| Generalisability | 21 | Discuss the generalisability (external validity) of the study results  Page 17 |
| Other information | | |
| Funding | 22 | Give the source of funding and the role of the funders for the present study and, if applicable, for the original study on which the present article is based  Page 19 |

*Give information separately for exposed and unexposed groups.

**Note:** An Explanation and Elaboration article discusses each checklist item and gives methodological background and published examples of transparent reporting. The STROBE checklist is best used in conjunction with this article (freely available on the Web sites of PLoS Medicine at http://www.plosmedicine.org/, Annals of Internal Medicine at http://www.annals.org/, and Epidemiology at http://www.epidem.com/). Information on the STROBE Initiative is available at www.strobe-statement.org.
