# Supplementary material for: Using the Cochrane Central Register of Controlled Trials to identify clinical trial registration is insufficient: a cross-sectional study
Source: BMC Med Res Methodol. 2020 Jul 25;20:200. doi: 10.1186/s12874-020-01083-y (PMC7382846; doi:10.1186/s12874-020-01083-y)
Supplement: Supplementary file 3 — Additional file 3: Appendix S2. Reference list of included Cochrane Reviews. [file 12874_2020_1083_MOESM3_ESM.docx]

1. Abiramalatha T, Thanigainathan S, Ninan B: Routine monitoring of gastric residual for prevention of necrotising enterocolitis in preterm infants. Cochrane Database Syst Rev 2019, 7:CD012937.

2. Al Said S, Alabed S, Kaier K, Tan AR, Bode C, Meerpohl JJ, Duerschmied D: Non-vitamin K antagonist oral anticoagulants (NOACs) post-percutaneous coronary intervention: a network meta-analysis. Cochrane Database Syst Rev 2019, 12:CD013252.

3. Annane D, Bellissant E, Bollaert PE, Briegel J, Keh D, Kupfer Y, Pirracchio R, Rochwerg B: Corticosteroids for treating sepsis in children and adults. Cochrane Database Syst Rev 2019, 12:CD002243.

4. Antequera Martin AM, Barea Mendoza JA, Muriel A, Saez I, Chico-Fernandez M, Estrada-Lorenzo JM, Plana MN: Buffered solutions versus 0.9% saline for resuscitation in critically ill adults and children. Cochrane Database Syst Rev 2019, 7:CD012247.

5. Barry T, Doheny MC, Masterson S, Conroy N, Klimas J, Segurado R, Codd M, Bury G: Community first responders for out-of-hospital cardiac arrest in adults and children. Cochrane Database Syst Rev 2019, 7:CD012764.

6. Bergman H, Buckley BS, Villanueva G, Petkovic J, Garritty C, Lutje V, Riveros-Balta AX, Low N, Henschke N: Comparison of different human papillomavirus (HPV) vaccine types and dose schedules for prevention of HPV-related disease in females and males. Cochrane Database Syst Rev 2019, 2019(11).

7. Best LM, Freeman SC, Sutton AJ, Cooper NJ, Tng EL, Csenar M, Hawkins N, Pavlov CS, Davidson BR, Thorburn D et al: Treatment for hepatorenal syndrome in people with decompensated liver cirrhosis: a network meta-analysis. Cochrane Database Syst Rev 2019, 9:CD013103.

8. Blessberger H, Lewis SR, Pritchard MW, Fawcett LJ, Domanovits H, Schlager O, Wildner B, Kammler J, Steinwender C: Perioperative beta-blockers for preventing surgery-related mortality and morbidity in adults undergoing non-cardiac surgery. Cochrane Database Syst Rev 2019, 9:CD013438.

9. Blessberger H, Lewis SR, Pritchard MW, Fawcett LJ, Domanovits H, Schlager O, Wildner B, Kammler J, Steinwender C: Perioperative beta-blockers for preventing surgery-related mortality and morbidity in adults undergoing cardiac surgery. Cochrane Database Syst Rev 2019, 9:CD013435.

10. Blumetti FC, Belloti JC, Tamaoki MJ, Pinto JA: Botulinum toxin type A in the treatment of lower limb spasticity in children with cerebral palsy. Cochrane Database Syst Rev 2019, 10:CD001408.

11. Burden S, Jones DJ, Sremanakova J, Sowerbutts AM, Lal S, Pilling M, Todd C: Dietary interventions for adult cancer survivors. Cochrane Database Syst Rev 2019, 2019(11).

12. Cooper TE, Hambleton IR, Ballas SK, Johnston BA, Wiffen PJ: Pharmacological interventions for painful sickle cell vaso-occlusive crises in adults. Cochrane Database Syst Rev 2019, 2019(11).

13. de Paiva CS, Pflugfelder SC, Ng SM, Akpek EK: Topical cyclosporine A therapy for dry eye syndrome. Cochrane Database Syst Rev 2019, 9:CD010051.

14. Downie LE, Ng SM, Lindsley KB, Akpek EK: Omega-3 and omega-6 polyunsaturated fatty acids for dry eye disease. Cochrane Database Syst Rev 2019, 12:CD011016.

15. Evans JR, Solomon AW, Kumar R, Perez A, Singh BP, Srivastava RM, Harding-Esch E: Antibiotics for trachoma. Cochrane Database Syst Rev 2019, 9:CD001860.

16. Flodgren G, O'Brien MA, Parmelli E, Grimshaw JM: Local opinion leaders: effects on professional practice and healthcare outcomes. Cochrane Database Syst Rev 2019, 6:CD000125.

17. Forget P, Borovac JA, Thackeray EM, Pace NL: Transient neurological symptoms (TNS) following spinal anaesthesia with lidocaine versus other local anaesthetics in adult surgical patients: a network meta-analysis. Cochrane Database Syst Rev 2019, 12:CD003006.

18. Franco JV, Turk T, Jung JH, Xiao YT, Iakhno S, Tirapegui FI, Garrote V, Vietto V: Pharmacological interventions for treating chronic prostatitis/chronic pelvic pain syndrome. Cochrane Database Syst Rev 2019, 10:CD012552.

19. Galvin IM, Levy R, Day AG, Gilron I: Pharmacological interventions for the prevention of acute postoperative pain in adults following brain surgery. Cochrane Database Syst Rev 2019, 2019(11).

20. Gibbs JC, MacIntyre NJ, Ponzano M, Templeton JA, Thabane L, Papaioannou A, Giangregorio LM: Exercise for improving outcomes after osteoporotic vertebral fracture. Cochrane Database Syst Rev 2019, 7:CD008618.

21. Hodder RK, O'Brien KM, Stacey FG, Tzelepis F, Wyse RJ, Bartlem KM, Sutherland R, James EL, Barnes C, Wolfenden L: Interventions for increasing fruit and vegetable consumption in children aged five years and under. Cochrane Database Syst Rev 2019, 2019(11).

22. Hoeg BL, Bidstrup PE, Karlsen RV, Friberg AS, Albieri V, Dalton SO, Saltbaek L, Andersen KK, Horsboel TA, Johansen C: Follow-up strategies following completion of primary cancer treatment in adult cancer survivors. Cochrane Database Syst Rev 2019, 2019(11).

23. Huber J, Stanworth SJ, Doree C, Fortin PM, Trivella M, Brunskill SJ, Hopewell S, Wilkinson KL, Estcourt LJ: Prophylactic plasma transfusion for patients without inherited bleeding disorders or anticoagulant use undergoing non-cardiac surgery or invasive procedures. Cochrane Database Syst Rev 2019, 11:CD012745.

24. Jaiswal N, Singh S, Agarwal A, Chauhan A, Thumburu KK, Kaur H, Singh M: Equivalent schedules of intradermal fractional dose versus intramuscular full dose of inactivated polio vaccine for prevention of poliomyelitis. Cochrane Database Syst Rev 2019, 12:CD011780.

25. Karjalainen TV, Jain NB, Heikkinen J, Johnston RV, Page CM, Buchbinder R: Surgery for rotator cuff tears. Cochrane Database Syst Rev 2019, 12:CD013502.

26. Kietpeerakool C, Rattanakanokchai S, Jampathong N, Srisomboon J, Lumbiganon P: Management of drainage for malignant ascites in gynaecological cancer. Cochrane Database Syst Rev 2019, 12:CD007794.

27. Kolkailah AA, Doukky R, Pelletier MP, Volgman AS, Kaneko T, Nabhan AF: Transcatheter aortic valve implantation versus surgical aortic valve replacement for severe aortic stenosis in people with low surgical risk. Cochrane Database Syst Rev 2019, 12:CD013319.

28. Lake JC, Victor G, Clare G, Porfirio GJ, Kernohan A, Evans JR: Toric intraocular lens versus limbal relaxing incisions for corneal astigmatism after phacoemulsification. Cochrane Database Syst Rev 2019, 12:CD012801.

29. Lewis SR, Pritchard MW, Fawcett LJ, Punjasawadwong Y: Bispectral index for improving intraoperative awareness and early postoperative recovery in adults. Cochrane Database Syst Rev 2019, 9:CD003843.

30. Lewis SR, Pritchard MW, Thomas CM, Smith AF: Pharmacological agents for adults with acute respiratory distress syndrome. Cochrane Database Syst Rev 2019, 7:CD004477.

31. Luger T, Maher CG, Rieger MA, Steinhilber B: Work-break schedules for preventing musculoskeletal symptoms and disorders in healthy workers. Cochrane Database Syst Rev 2019, 7:CD012886.

32. Merriel A, Ficquet J, Barnard K, Kunutsor SK, Soar J, Lenguerrand E, Caldwell DM, Burden C, Winter C, Draycott T et al: The effects of interactive training of healthcare providers on the management of life-threatening emergencies in hospital. Cochrane Database Syst Rev 2019, 9:CD012177.

33. Muelbert M, Lin L, Bloomfield FH, Harding JE: Exposure to the smell and taste of milk to accelerate feeding in preterm infants. Cochrane Database Syst Rev 2019, 7:CD013038.

34. Ng DHC, Klassen JR, Embleton ND, McGuire W: Protein hydrolysate versus standard formula for preterm infants. Cochrane Database Syst Rev 2019, 7:CD012412.

35. O'Hara L, Smith ER, Barlow J, Livingstone N, Herath NI, Wei Y, Spreckelsen TF, Macdonald G: Video feedback for parental sensitivity and attachment security in children under five years. Cochrane Database Syst Rev 2019, 11:CD012348.

36. Palmer JS, Monk AP, Hopewell S, Bayliss LE, Jackson W, Beard DJ, Price AJ: Surgical interventions for symptomatic mild to moderate knee osteoarthritis. Cochrane Database Syst Rev 2019, 7:CD012128.

37. Pantoja T, Grimshaw JM, Colomer N, Castanon C, Leniz Martelli J: Manually-generated reminders delivered on paper: effects on professional practice and patient outcomes. Cochrane Database Syst Rev 2019, 12:CD001174.

38. Quigley M, Embleton ND, McGuire W: Formula versus donor breast milk for feeding preterm or low birth weight infants. Cochrane Database Syst Rev 2019, 7:CD002971.

39. Regnaux JP, Davergne T, Palazzo C, Roren A, Rannou F, Boutron I, Lefevre-Colau MM: Exercise programmes for ankylosing spondylitis. Cochrane Database Syst Rev 2019, 10:CD011321.

40. Romantsik O, Bruschettini M, Moreira A, Thebaud B, Ley D: Stem cell-based interventions for the prevention and treatment of germinal matrix-intraventricular haemorrhage in preterm infants. Cochrane Database Syst Rev 2019, 9:CD013201.

41. Safi S, Sethi NJ, Nielsen EE, Feinberg J, Jakobsen JC, Gluud C: Beta-blockers for suspected or diagnosed acute myocardial infarction. Cochrane Database Syst Rev 2019, 12:CD012484.

42. Schnabel A, Reichl SU, Weibel S, Zahn PK, Kranke P, Pogatzki-Zahn E, Meyer-Friessem CH: Adductor canal blocks for postoperative pain treatment in adults undergoing knee surgery. Cochrane Database Syst Rev 2019, 2019(10).

43. Scott AM, Clark J, Julien B, Islam F, Roos K, Grimwood K, Little P, Del Mar CB: Probiotics for preventing acute otitis media in children. Cochrane Database Syst Rev 2019, 6:CD012941.

44. Steed L, Sohanpal R, Todd A, Madurasinghe VW, Rivas C, Edwards EA, Summerbell CD, Taylor SJ, Walton RT: Community pharmacy interventions for health promotion: effects on professional practice and health outcomes. Cochrane Database Syst Rev 2019, 12:CD011207.

45. Storebo OJ, Elmose Andersen M, Skoog M, Joost Hansen S, Simonsen E, Pedersen N, Tendal B, Callesen HE, Faltinsen E, Gluud C: Social skills training for attention deficit hyperactivity disorder (ADHD) in children aged 5 to 18 years. Cochrane Database Syst Rev 2019, 6:CD008223.

46. Sweeney C, Ryan F, Ledwidge M, Ryan C, McDonald K, Watson C, Pharithi RB, Gallagher J: Natriuretic peptide-guided treatment for the prevention of cardiovascular events in patients without heart failure. Cochrane Database Syst Rev 2019, 10:CD013015.

47. Treanor CJ, Santin O, Prue G, Coleman H, Cardwell CR, O'Halloran P, Donnelly M: Psychosocial interventions for informal caregivers of people living with cancer. Cochrane Database Syst Rev 2019, 6:CD009912.

48. Wrzosek A, Jakowicka-Wordliczek J, Zajaczkowska R, Serednicki WT, Jankowski M, Bala MM, Swierz MJ, Polak M, Wordliczek J: Perioperative restrictive versus goal-directed fluid therapy for adults undergoing major non-cardiac surgery. Cochrane Database Syst Rev 2019, 12:CD012767.

49. Yamamoto S, Yamaga T, Nishie K, Nagata C, Mori R: Positive airway pressure therapy for the treatment of central sleep apnoea associated with heart failure. Cochrane Database Syst Rev 2019, 12:CD012803.

50. Zeng L, Yu X, Yu T, Xiao J, Huang Y: Interventions for smoking cessation in people diagnosed with lung cancer. Cochrane Database Syst Rev 2019, 6:CD011751.
